# Bibliography

**Published:** 1887-04

**Authors:** 


### BIBLIOGRAPHY.

A Practical Treatise on Obstetrics. Vol. I. (4 vols.), Anatomy of the Internal and External Genitals, Physiological Phenomena (Menstruation and Fecundation). By A. Charpentier, M. D., Paris. Illustrated with lithographic plates and wood engravings. This is also Vol. I. of the "*Cyclopedia of Obstetrics and Gynecology*" (12 vols.), issued monthly during 1887. New York: William Wood & Company.

A Practical Treatise on Obstetrics. Vol. II. (4 vols.), The Pathology of Pregnancy. By A. Charpentier, M. D., Paris. Illustrated with lithographic plates and wood engravings. This is also Vol. II. of the "*Cyclopedia of Obstetrics and Gynecology*" (12 vols.), issued monthly during 1887. New York: William Wood & Company.

This is a translation from the French edition of 1882 made under the supervision of Egbert H. Grandin, M. D., Obstetric Surgeon to the New York Maternity Hospital; Instructor in Gynæcology at the New York Polyclinic; Fellow of the Obstetrical Society, etc.

It is claimed by the author that although the country is liberally supplied with works on obstetrics, "the classical treatises are no longer exponents of the modern science." We do not know exactly upon what he bases the assertion; for we have Lusk's last edition, which certainly is an "exact exponent of the science" from an American stand-point. But, as a matter of fact, no text book can show the very latest phases of treatment, so progressive are the Americans in that line; this can only be had by reading the *Medical Journals*; they are to the text books what the daily is to the weekly newspaper. Professor Charpentier being at the head of the obstetric clinic at the Paris School of Medicine and adjunct to the chair of obstetrics (Professors Pajot & Depaul) was in position to keep well abreast of the progress in this science in France, in theory and in practice. No doubt it contains many new and valuable hints. We have not yet had opportunity to give the two volumes, with which the publishers have favored us, more than a cursory examination. The get up is fully equal to Messrs. Wm. Wood & Co.'s usual superior style. Cloth: pp 506. The series of twelve is to be sold for the nominal price of $16.50 in three payments. It is certainly a valuable collection and profusely illustrated.

---

MANUAL OF TREATMENT. A Concise Presentation of the Modern Methods of Treating Diseases employed by the best Authors, Teachers and Practitioners; arranged with special reference to the needs of American practitioners, by C. F. Taylor, M. D., editor of the *Medical World*, and W. F. Waugh, M.D., A.M., Professor of Practice and Clinical Medicine in the Medico-Chirurgical College, of Philadelphia; Physician to the Medico-Chirurgical Hospital of Philadelphia; member American Academy of Medicine; Academy of Natural Sciences; Microscopical and Biological Section; Vice President of the Medico-Segal Society of Philadelphia; Late of U. S. Navy, etc., etc., published by THE MEDICAL WORLD, 1520 Chestnut street, Philadelphia, 1887. Cloth, 530 pages, price $4.00.

Dr. Taylor begun the preparation of this work in 1885, but was compelled to abandon it for want of time. Later he met Professor Waugh, who being impressed as Dr. Taylor was with the need of such a work—in order to save the general practitioner much valuable time which would be required to make a thorough research through a great mass of literature, both foreign and domestic—became interested in it, and the combined labors of both these gentlemen have given the profession one of the most useful books, practically, that we have seen. The nature of Professor Waugh's occupation and his taste, naturally lead him to explore the general field of literature, and in so doing he culled the best items of treatment of prominent and successful practitioner, not only in America, but in all parts of Europe, and though the work is designed for the American practitioner, and is made up largely of the more popular methods of American treatment, and especially that of certain distinguished physicians, it is by no means confined to such.

We are much pleased with it. Being arranged alphabetically it is very handy for reference; is really a "manual." It shows in its preparation not only deep and thorough research, but a wise discrimination—good judgment in selection.

The mechanical execution is highly creditable to the MEDICAL WORLD. Price, in fine muslin and gilt, only $4.00. Should you order the book—and we would advise you to do so—please mention DANIEL'S TEXAS MEDICAL JOURNAL.

